# The Landscape of Primary Central Nervous System Lymphoma (PCNSL): Clinicopathologic and Genomic Characteristics and Therapeutic Perspectives

**DOI:** 10.3390/cancers17172909

**Published:** 2025-09-04

**Authors:** Huijuan Jiang, Lin Nong

**Affiliations:** 1 Department of Hematology, Tianjin Medical University General Hospital, Tianjin Key Laboratory of Bone Marrow Failure and Malignant Hemopoietic Clone Control, Tianjin Institute of Hematology, Tianjin 300052, China; 2 Department of Pathology, National Cancer Center/National Clinical Research Center for Cancer/Cancer Hospital, Chinese Academy of Medical Sciences and Peking Union Medical College, Beijing 100021, China

**Keywords:** primary central nervous system lymphoma, large B-cell lymphoma, pathophysiology, molecular profiling, diagnosis, immunotherapy, targeted therapy, novel therapy

## Abstract

Primary central nervous system lymphoma (PCNSL) is a rare extra-nodal non-Hodgkin lymphoma confined to the central nervous system. The cancer biology of PCNSL remains incomplete and is often associated with genetic aberrations with abnormal signaling pathways, cell differentiation, regulation of epigenetic modification, and the tumor microenvironment. Stereotactic brain biopsy remains the gold standard for the diagnosis of PCNSL. For patients ill-suited for biopsy, MYD88 and IL-10 may be important biomarkers to diagnose PCNSL. High-dose methotrexate-based polychemotherapy is currently the standard induction treatment for PCNSL, followed by consolidation treatments including autologous stem cell transplant and whole-brain radiotherapy. Some studies suggest that low-dose lenalidomide is recommended as a maintenance therapy for PCNSL. Currently, relapse rates of PCNSL range from 25 to 50% with poor prognosis. Insight research is necessary to identify novel targeted treatments to improve outcomes in relapsed/refractory disease, such as immunomodulatory drugs, immune checkpoint inhibitors, signaling pathway inhibitors, and chimeric antigen receptor T-cell therapy.

## Simple Summary

Primary central nervous system lymphoma (PCNSL) is a rare, aggressive lymphoma confined to the central nervous system that is distinct from systemic diffuse large B-cell lymphomas. The pathophysiology of PCNSL remains incompletely understood, and patient outcomes are less favorable compared to lymphomas outside the CNS. Standard first-line induction treatment involves high-dose methotrexate-based polychemotherapy, followed by consolidation with autologous stem cell transplantation and whole-brain radiotherapy. Despite advances in multimodal therapy, treatment failure and relapses remain common. This review provides an overview of current understanding of PCNSL, addressing recent insights into pathogenesis, diagnostic advancements, current treatment strategies, and emerging novel targeted treatments and immunotherapies.

## 1. Introduction

Primary central nervous system lymphoma (PCNSL) is a rare extra-nodal non-Hodgkin lymphoma confined to the central nervous system (CNS) that involves the brain, leptomeninges, and spinal cord. Up to 90% of PCNSL are diffuse large B-cell lymphomas (DLBCLs), with most expressing a non-Germinal Center B-cell-like (non-GCB) phenotype [1,2,3]. In the fifth edition of the World Health Organization (WHO) classification of hematolymphoid tumors, PCNSL is classified as a subtype of primary large B-cell lymphomas of immune-privileged sites, together with primary testicular diffuse large B-cell lymphoma and vitreoretinal large B-cell lymphoma [3]. Most cases of PCNSL occur in immunocompetent patients and are typically unrelated to Epstein–Barr virus (EBV) infection. However, there is an increasing incidence of PCNSL (~3%) in immunocompromised patients who are EBV-positive [2,4,5]. Up to 70% of PCNSL exhibits molecular features of the MCD group, with a high mutation frequency of MYD88 and CD79B. MYD88, together with TLR9 and the B-cell receptor (BCR), forms the My-T-BCR supercomplex, drives constitutive NF-κB activation, and prevents apoptosis of lymphoma cells [6,7].

The overall prognosis of PCNSL is poor despite intense chemotherapy protocols, mainly due to the high tendency of relapse [8]. Studies on PCNSL are still limited because of its low incidence and difficulty in obtaining samples for research. Recent advances in understanding the molecular profiles of PCNSL have improved the diagnosis and development of potential therapeutic strategies.

## 2. Pathogenesis and Genetic Alterations

The CNS is a special organ system that normally lacks regular lymphoid tissue. The source of B-cells that develop into PCNSL and the mechanism for their entry into the CNS, despite the protection conferred by the blood–brain barrier (BBB), remain unconfirmed, which contributes to the disease’s characteristic clinical manifestation and biological behavior [9,10]. It is suggested that self/polyreactive B-cells are the cellular origin of PCNSL, and that the deregulation of B-cell differentiation plays a crucial role in its pathogenesis.

PCNSL displays molecular features of the activated B-cell-like (ABC) subgroup, mostly with a “C5”, “MCD”, or “MYD88-like” cluster according to the molecular classification of DLBCL [2,3,11,12,13]. Genetic aberrations associated with PCNSL include somatic mutations in genes encoding proteins or receptors involved in signaling pathways, the cell cycle, cell differentiation, or the regulation of epigenetic modifications. Other genetic alterations include recurrent amplifications and deletions, non-coding RNAs, and microRNAs (miRNA). The tumor microenvironment (TME) may also play a role in the pathogenesis of PCNSL [14,15]. Although PCNSL shares many similar genetic alterations with the systemic DLBCL ABC subtype with an MCD cluster, PCNSL should be distinguished clearly from systemic DLBCL according to its specific combination of genetic aberrations and distinct expression profiles [16].

### 2.1. Genetic Mutations and Signaling Pathway Modulation

The genetic events that occur in PCNSL have been investigated with high-throughput sequencing and multi-omics profiling technologies. PCNSL has well demonstrated a high frequency of somatic nonsynonymous mutations in genes such as MYD88 (mostly L265P) and CD79B (mostly Y196), which usually occur simultaneously at a rate of around 60–70%, accompanied by mutations in PIM1 [1,2,17,18,19,20,21]. The mutation frequency of MYD88, CD79B, and PIM1 in PCNSLs is much higher than that of systemic DLBCL (67%, 63%, 70% versus 17%, 11%, 26%) [2,22]. A total of 67% of PCNSL cases were classified as MCD, while fewer than 15% of systemic DLBCL cases were classified as MCD [2,22]. Other genes that are involved in PCNSL include CARD11, PRDM1, TBL1XR1, TNFAIP3, B2M, CDKN2A, KMT2D, TERT, KLHL14, ETV6, CD58, CIITA, etc. Somatic hypermutation (SHM) involved in PCNSL affects genes including IGHV4–34, BTG2, H1–4, KLHL14, MYC, PAX5, PIM1, RHOH, and SUSD2 [2,17,19,20,23]. Ultimately, the accumulation of numerous mutations and their complex regulatory effects on signaling pathways results in tumorigenesis.

Genomic investigations indicate that several signaling pathways participate in the cell proliferation and survival of lymphoma cells in PCNSL, particularly constitutive NF-κB activation and deregulated TLR, BCR, PI3K/AKT/mTOR, and JAK-STAT pathways leading to cell survival and proliferation [18,20,24]. The above-described signaling pathways are modulated by intricate mutated genes in PCNSL pathogenesis. MYD88 L265P integrates with CD79B to foster NF-κB activation via TLR and BCR signaling. These genetic abnormalities eventually block terminal B-cell differentiation, deregulate the cell cycle, prevent apoptosis, and promote immune evasion of B-cells [17,18]. Pindzola et al. have proposed aberrant spontaneous splenic GCBs as a likely cell of origin for MCD DLBCL [6]. Despite the cells not exhibiting a germinal center (GC) profile, several aberrant somatic mutations were identified in PCNSL. The My-T-BCR supercomplex prevents the B-cells from apoptosis and grants them a survival advantage in the CNS. The B-cells undergo somatic hypermutation (SHM) in the GC reaction and acquire oncogenic hits, and finally, malignant transformation occurs. The abnormal B-cells become neoplastic B-cells, and the terminal differentiation is blocked, suggesting that PCNSL is derived from a late GCB cell [25,26,27,28]. Several other somatic mutations are engaged in this process. For instance, an inactivating mutation of PRDM1 and an activating mutation of CARD11 may induce the activation of the NF-κB and BCL6 translocation [18,29]. Inactivating PRDM1 mutations also contribute to impaired IG class-switch recombination and deregulated B-cell terminal differentiation [18,30,31]. TBL1XR1 mutations, which occur in about 40% of PCNSL, lead to impaired plasma cell differentiation [2,17,18,32]. TBL1XR1 mutations and BCL6 disruption may block the evolution of GCB cells and retain B-cells in an immature memory B-cell phase [17,18]. 

Compared to systemic DLBCL, PCNSL has a lower rate of TP53 mutations and lower rate of MYC/BCL2 rearrangements but more frequent BCL6 translocations. Mutations in genes encoding epigenetic regulators such as EZH2, CREBBP, and EP300 are usually absent in PCNSL [24,33,34]. Moreover, genetic alterations in immune evasion-related genes HLA and CD274/PDCD1LG2 arise as late genetic events [35].

According to some clinical data, signaling pathway mutations might be associated with the therapeutic response and prognosis of PCNSL. Through whole-exome sequencing (WES), the incidence of RTK-RAS and PI3K pathway mutations was higher in durable clinical benefit patients, while that of Notch and Hippo pathway mutations was higher in no durable benefit patients [36].

### 2.2. Epigenetic Modifications

Epigenetic modifications that may also participate in PCNSL pathogenesis include hypermethylation of DAPK (84%), CDKN2A (75%), MGMT (52%), and RFC (30%), with prospective therapeutic value [25]. Silencing of other genes through methylation, such as KMT2D, GSTP1, TIMP-3, p16(INK4A), and RB1, has also been reported in PCNSL [37]. 

### 2.3. Recurrent Amplifications and Deletions

Recurrent amplifications involve 18q21.23, 19p13.13, 1q32.1, 11q23.3, 2q22.3, 3q12.3, 9p24.1, and 12p [17,38,39,40]. Recurrent deletions involve 6q21, 6p21, 6q27, 9p21.3, 6p25.3, 22q11.22, and 14q32.33 [17,38,39,40]. Among these frequent copy number alterations (CNVs), while 3q12.3 gains induce overexpression of NFKBIZ and activate the NF-κB pathway, 6q21 losses lead to the loss of PRDM1 and TNFAIP3, which may deregulate B-cell terminal differentiation and also activate the NF-κB pathway. Losses of 9p21.3 harboring CDKN2A may lead to deregulated cell cycle activity, increased chromosomal instability, and proliferative activity [32,41,42].

Structural variants at 9p24.1 (affecting CD274/PD-L1 and PDCD1LG2/PD-L2, with copy number gains in 67% of cases and chromosomal translocations in 13% of cases) and at 6p21.32 (heterozygous deletions, harboring the MHC class II encoding genes HLA-DRB, HLA-DQA, HLA-DQB) may contribute to TME of PCNSL [24,43]. Compared to PCNSL, the most common cytogenetic alterations affecting PD-L1/PD-L2 locus in systemic DLBCL are copy number gains (13%), followed by translocations and amplifications, and the majority of them occur in non-GCB cases [43,44]. Furthermore, upregulation of miRNA has been reported in PCNSL, such as miR-17-5p, miR-155, miR-21, and miR-196b, but their role in the pathogenesis of PCNSL remains unclear [45,46,47].

### 2.4. Microenvironments

The TME in PCNSL is distinct in that lymphoma cell infiltration around blood vessels is commonly shown in most PCNSL cases [48], whereas CD8+ T-cells are mostly within the tumor [49]. Monocytes and macrophages also participate in PCNSL pathogenesis. M1-like macrophages (CD68+CD163low) play an antitumoral role, whereas M2-like macrophages (CD68+CD163high) usually have protumoral effects, are predominantly found within the tumor, and express immune-checkpoint-related molecules such as PD-L1 and TIM3, conferring an immunosuppressive TME to PCNSL [50]. Inactivation of CD58 and B2M through mutations and deletions contributes to escape from immune surveillance in DLBCL. Inactivated B2M leads to lack of HLA-I and escape from lysis by cytotoxic T lymphocytes, while loss of function of CD58 may result in decreased recognition of lymphoma cells by both T-cells and NK cells [51]. Similarly, somatic mutations, deletions, or rearrangements of B2M, CD58, and CIITA also play roles in immune escape in PCNSL [39,52,53,54]. 

With the development of single-cell technologies and the continued improvement of spatial transcriptome technologies, more studies will focus on the characteristics of the TME in PCNSL. Although the CNS is an immune-privileged region, BBB permeability increases after tumor occurrence, resulting in a high TME heterogeneity similar to lymphomas outside the CNS [14,55]. A study of a comprehensive multi-omics analysis classified PCNSLs into four robust and prognostically significant clusters (CS) [54]. CS1 and CS2 presented with an immune-cold hypermethylated profile with distinct clinical behavior. The “immune-hot” CS4 group was enriched with mutations increasing JAK-STAT and NF-κB pathway activity and had the most favorable clinical outcome. The heterogeneous-immune CS3 group had the worst prognosis, due to its association with enriched HIST1H1E mutations and meningeal infiltration. Thus, the use of targeted therapies, such as cyclin D-CDK4,6 plus PI3K inhibitors for CS1, lenalidomide/demethylating drugs for CS2, EZH2 inhibitors for CS3, and immune checkpoint inhibitors/JAK1 inhibitors for CS4, may have great potential [54,56]. Similarly, Xia et al. defined four TME types based on the degree of T-cell infiltration, including “hot”, “invasive margin excluded (IME)”, “invasive margin immunosuppressed (IMS)”, and “cold” [14]. They identified the TME remodeling pattern and immune pressure-sensing model and revealed the developmental sequence and function correlation of the different TME patterns [14]. They found that IMS is a transitional state after the hot state, while both IME and the cold state are terminal states of tumor progression with differing cell fates based on T-cell load. Tumor cells often enter the cold state when there is a low infiltration of immune killing T-cells to conduct high proliferation and expansion, but enter IME when T-cells are heavily infiltrating and form a junction between immune cells and IME. The expression of immune checkpoint molecules and receptors is spatially heterogeneous. PD-L1 expression is distributed throughout the hot state, concentrated at the invasive margin of tumor cells and immune cells in IMS, and gradually decreased in IME and the cold state. These findings indicate that it is important to clarify the TME state for the use of immune checkpoint molecule inhibitors and CAR-T therapy for PCNSL treatment [14].

Previous studies have shown that TME might be related to the prognosis of PCNSL and other immune-privileged large B-cell lymphomas (IP-LBCLs). In PCNSL, elevated CD4⁺ and CD8⁺ T-cell levels and M1 macrophage infiltration were correlated with improved progression-free survival (PFS) [57].

## 3. Diagnosis

### 3.1. Epidemiology and Clinical Presentation

PCNSL accounts for 2–3% of all brain malignancies and less than 1% of all non-Hodgkin lymphomas, with a higher incidence rate in elderly patients over 60 years old and a slight male predominance (male-to-female ratio of 3:2) [1,58]. Patients suffering from PCNSL may initially lack specific symptoms or signs of disease compared to other central nervous system disorders. Headache, focal neurological deficits, cognitive disturbances, and neuropsychiatric symptoms are the most common symptoms in PCNSL patients [59,60,61,62]. The local neurological symptoms depend on the tumor site. Brain imaging techniques (such as brain computed tomography (CT) or magnetic resonance imaging (MRI)) in patients with neurologic symptoms or signs usually show intracranial space-occupying lesions.

### 3.2. Imaging Examinations

**Figure 1:**
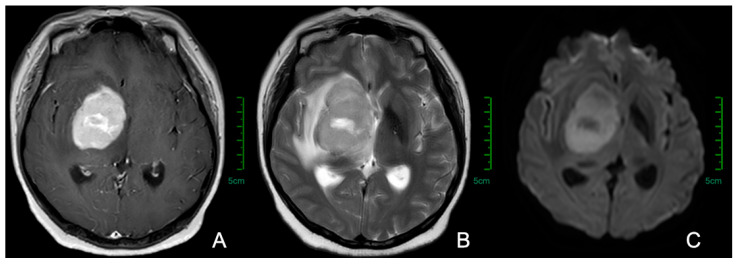
Characteristic imaging features of PCNSL on MRI. Magnetic resonance images from a patient with primary central nervous system lymphoma. (**A**) Contrast-enhanced T1-weighted sequence demonstrates homogeneous enhancement of the tumor in the right basal ganglia region. (**B**) T2-weighted sequence shows hyperintense signal surrounding the tumor, demonstrating vasogenic cerebral edema. (**C**) Diffusion-weighted imaging (DWI) demonstrates restricted diffusion within the lesions.

More than one-third of the PCNSL lesions are localized to the cerebral hemispheres, followed by the thalamus/basal ganglia, corpus callosum, periventricular region, cerebellum, and a minority in the leptomeninges [60,63,64]. Currently, craniocerebral enhanced MRI is the predominant diagnostic tool to evaluate potential therapeutic efficacy against PCNSL. Lesions in PCNSL may be solitary or multifocal, with more than 50% being solitary. The enhanced MRI images can show space-occupying lesions with varying degrees of enhancement, vasogenic edema, and restricted diffusion (Figure 1). 

If patients are not fit to receive enhanced MRI, they may undergo non-enhanced MRI, CT with or without contrast, or fluorodeoxyglucose (FDG)–positron emission tomography (PET). The role of PET brain imaging has not been established yet [65]. The International Primary CNS Lymphoma Collaborative Group (IPCG) recommends a systemic staging evaluation with 18F-FDG-PET [66]. The FDG-PET of PCNSL shows an avid lesion with homogeneous uptake in high glucose metabolism, which is 2–3 times more than in normal tissue [62,67]. In addition, a total-body PET scan can distinguish PCNSL from secondary central nervous system lymphoma (SCNSL) [68]. However, due to the high glucose metabolism level in brain parenchyma, the application of 18F-FDG in PCNSL diagnosis is limited to some extent. Some novel molecular and functional tracers, like the CXCR4-directed PET tracer 68Ga-pentixafor [69] and 18 F-Fludarabine [70], have been explored for use in diagnosing PCNSL.

### 3.3. Pathology and Cerebrospinal Fluid (CSF) Examinations

**Figure 2:**
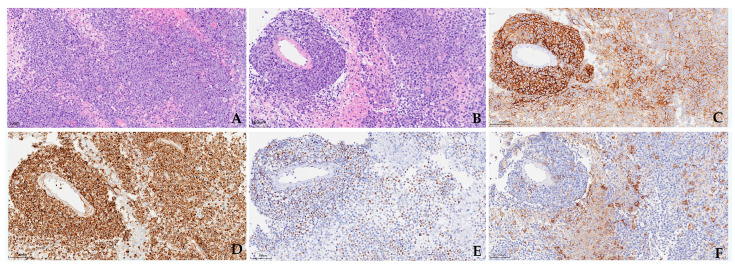
Pathological features and typical immunophenotype of PCNSL. Medium to large neoplastic cells diffusely infiltrate the neural parenchyma (**A**) with perivascular accumulation (**B**). The tumor cells strongly express CD20 (**C**) with a mostly non-GCB phenotype and double expression of BCL-2 (**D**) and c-Myc (**E**). The variable expression of PD-L1 (**F**) indicates a heterogeneous TME status.

Brain biopsy by stereotactic biopsy remains the gold standard for the diagnosis of PCNSL. Up to 90% of PCNSL are DLBCL. Other less common categories include low-grade B-cell, mantle cell, Burkitt, lymphoblastic, and peripheral T-cell lymphomas [1,71]. Histologically, primary CNS DLBCL consists of medium to large atypical centroblastic or immunoblastic cells, which usually accumulate within the perivascular space or diffusely infiltrate the neural parenchyma. Geographical necrosis and a background of mixed inflammatory cells can also be seen [1,17]. The tumor cells express pan B-cell markers and show a non-GCB phenotype, mostly CD10-/BCL6+/MUM1+. The Ki-67 proliferative index is usually high (>70% or even >90%) [1]. Up to 80% cases have a BCL-2 and c-Myc double-expressor phenotype, but are mostly not related to a BCL-2 and MYC rearrangement. The incidence of double-hit lymphoma with MYC/BCL-2 translocation is approximately 1.3% [9,11,62,72]. The expression of PD-L1 was found in 30% of PCNSL cases [25,61]. The pathological and immunohistochemical features of a representative case of PCNSL are shown in Figure 2. The cell origin of PCNSL is speculated to be late germinal center B-cells or long-lived memory B-cells, with blocked terminal B-cell differentiation [1,17,73]. In recent years, efforts have been made to develop a deep learning model to precisely distinguish PCNSL from other CNS tumors [74].

If patients are not fit for stereotactic biopsy, CSF detection can be used to facilitate the diagnosis of PCNSL, such as cytology, flow cytometry (FCM), immunoglobulin heavy chain gene rearrangement testing, circulating tumor DNA (ctDNA), miRNA, and cytokine concentrations. Up to 20–30% of PCNSL patients may have positive results from CSF cytology and immunophenotyping with FCM [61,75,76,77,78,79]. MYD88-gene mutation and increased interleukin-10 (IL-10) levels in CSF have been observed in PCNSL, and they show promising sensitivity (94–98%) and specificity (98–99%) for both newly diagnosed and recurring PCNSL [80,81]. It was reported that the combined detection of MYD88 and IL-10 in CSF was an important biomarker to improve the timely recognition of PCNSL [78,82]. In a recent clinical trial, the researchers found that baseline levels of interferon-α (IFN-α) and the IL10/IL6 ratio in CSF emerged as potential predictors of PFS in PCNSL [36]. 

According to some studies, the ctDNA in plasma or CSF might be a valuable noninvasive biomarker for diagnosis and an independent prognostic factor for clinical outcomes in PCNSL patients [83,84]. The detection of miRNA (like miR-16-5p, miR-21-5p, miR-92a-3p, and miR-423-5p) in CSF can effectively demonstrate the genetic makeup of CNS tumors, where their roles remain unclear [76,85,86]. However, these CSF examinations are insufficient to justify a definitive diagnosis and still need to be studied in more prospective trials. If a brain biopsy cannot be performed, the diagnosis of PCNSL can only be made through CSF when positive results are observed in combination with the detection of typical clinical and imaging characteristics. 

## 4. Prognosis

The prognosis of PCNSL is less favorable than systemic DLBCL or other extranodal DLBCL of non-immune-privileged sites but has a trend to be better than secondary CNS lymphoma [87,88]. The 5-year overall survival rate of PCNSL is 30~40%, while it is about 70% in DLBCL after R-CHOP therapy. The median PFS (mPFS) and the median OS (mOS) of PCNSL were 10.5 months and 25.3 months, respectively [89]. However, modern immunochemotherapy and consolidation treatments such as autologous transplantation may help a portion of younger patients (15–20%) achieve long-term survival. It was reported that the 5-year OS rate of patients treated with first-line hematopoietic cell transplant-autologous stem cell transplant (HCT-ASCT) could reach 76%. Increasing age, elevated LDH, high IPI scores, high CSF concentration, and deep brain involvement are associated with inferior prognosis [90,91]. A low M1-to-M2 ratio of macrophages was also reported to be associated with poor outcomes [50].

Increasing evidence shows that gene mutations are closely associated with the prognosis of PCNSL. It has been demonstrated that several gene alterations are related to a poorer prognosis in PCNSL patients, including deletions or somatic mutations of HLA genes, mutations of CD79B and GNA13, high phosphorylated STAT6 expression, and a high level of CNVs [72,92]. Yuan et al. found that TMSB4X mutation and a high expression of TMSB4X protein were associated with inferior overall survival (OS) and established a prognostic risk scoring system, which includes Karnofsky performance status and six mutated genes (BRD4, EBF1, BTG1, CCND3, STAG2, and TMSB4X) [40].

In older PCNSL patients, the outcomes have been optimized with HD-MTX combination induction and maintenance therapy, but more factors could affect prognosis. According to a multicenter retrospective study, the 3-year PFS rate and OS rate were 30–65% and 47–84%, respectively [93]. The Cooperative Oncology Group (ECOG) performance status (PS), age, hypoalbuminemia, and higher CIRS-G score all adversely affect OS.

## 5. Treatments

Untreated PCNSL patients usually die within 1–3 months [62]. Since untreated PCNSL patients have a very poor prognosis, treatments should be initiated as soon as the diagnosis is confirmed. Treatments of PCNSL include two stages: induction and consolidation treatment. High-dose methotrexate (HD-MTX)-based chemotherapy is currently the standard induction treatment. Consolidation treatments include ASCT and whole-brain radiotherapy (WBRT). According to the European Association of Neuro-Oncology (EANO) guidelines for the treatment of PCNSL, patients with a large space-occupying lesion with acute symptoms of brain herniation can receive surgical resection to rapidly reduce intracranial pressure. However, for patients with unifocal and resectable lesions suspected of PCNSL, surgical resection data are limited [94].

### 5.1. Induction Treatments

Intravenous HD-MTX (1–8 g/m2) is the main drug in induction treatments for PCNSL. According to several recent studies, a combination of HD-MTX with other BBB-penetrating agents (like thiotepa, carmustine, temozolomide (TMZ), etc.) was shown to be more effective than an HD-MTX monotherapy [94,95]. However, there is no consensus on a first-line induction treatment for PCNSL. Based on a phase II study, the MATRix therapy (combining HD-MTX, cytarabine, thiotepa, and rituximab) showed the best survival rates based on a phase II study, with an overall response rate (ORR) of 87% and a complete remission rate (CRR) of 49% [96]. The IELSG32 randomized trial showed that PCNSL patients treated with MATRix and consolidation treatment (ASCT or WBRT) had a 7-year OS rate of 70% [97]. Since the sensitivity to first-line treatment is considered an independent prognostic factor for improved OS, more randomized controlled trials (RCTs) are needed to develop better induction treatments in PCNSL.

In recent years, combination treatments with small-molecule targeted agents have also been explored. In previous studies, the expression of programmed cell death-1 (PD-1) in tumor-infiltrating lymphocytes (TILs) and PD-L1 in tumor cells was higher than in systemic DLBCL and was associated with prognosis in PCNSL. The PD-1 pathway has emerged as a potential therapeutic target [57,98]. In a phase 2 study (ChiCTR1900027433), sintilimab (anti-PD-1 antibody) combined with HD-MTX, TMZ, and rituximab (SMTR regimen) has shown good responses in newly diagnosed PCNSL. The ORR was 96.3% with 92.6% (25/27) of CRR. At a median follow-up of 24.4 months, the median duration of response (mDOR), median PFS (mPFS), and median OS (mOS) values were not reached [36]. In a prospective phase II trial, the combination of rituximab, HD-MTX, and orelabrutinib (Bruton’s tyrosine kinase inhibitor) (R-MO regimen) showed good outcomes and favorable safety in the first-line treatment of PCNSL. The best ORR was 97.1% and the best CRR rate was 94.1% with a 1-year PFS rate of 83.6% [99]. In the phase IB part of the LOC-R01 study, the R-MPV regimen (rituximab, methotrexate, procarbazine, vincristine, and prednisone) in combination with lenalidomide (LEN) or ibrutinib (Bruton’s tyrosine kinase inhibitor) was tested in newly diagnosed PCNSL. The early results showed that the ORR levels were 76.9% and 83.3% in the LEN and ibrutinib arm, respectively. The phase II part of the study is ongoing [100].

### 5.2. Consolidation and Maintenance Treatments

If induction treatments demonstrate at least a partial response (PR) (a 50% reduction in tumor mass compared to baseline), PCNSL patients can receive consolidation treatment, including high-dose chemotherapy, ASCT, or WBRT, etc. Based on a phase II study, WBRT and ASCT are effective consolidation treatments for PCNSL patients aged ≤60 years. However, cognitive impairment was observed after WBRT but might be preserved or improved after ASCT [97,101]. Additionally, ASCT had less neurocognitive toxicity and was more effective at preventing relapse compared to WBRT (2-year progression-free survival (PFS) of 87% vs. 63%) but had a higher treatment-related mortality (about 10%) [102]. In a prospective, single-arm, phase 2 trial with high-dose chemotherapy (induction treatment consisted of HD-MTX, HD-cytarabine, and rituximab) followed by ASCT in patients aged ≥65y, fit patients with newly diagnosed PCNSL achieved a 12-month PFS of 58.8% [103]. It indicated that high-dose chemotherapy and ASCT are effective in certain selected older PCNSL patients. Thus, ASCT is currently recommended as a consolidation treatment for PCNSL patients below 70 years who are in acceptable clinical condition (KPS>70). 

For ASCT, there is no consensus on the optimal conditioning regimens in PCNSL patients. In an observational study, the outcomes in PCNSL patients undergoing ASCT with the three most commonly used conditioning regimens were assessed: TBC (thiotepa, busulfan, and cyclophosphamide), TT-BCNU (thiotepa and carmustine), and BEAM (carmustine, etoposide, cytarabine, and melphalan) [104]. The results showed that a thiotepa-based conditioning regimen compared to BEAM had higher survival rates (adjusted PFS rate 75% vs. 58%) and a lower relapse risk but higher rates of early toxic effects and non-relapse mortality (NRM) compared to other regimens. In a retrospective study to compare thiotepa-based conditioning regimens (thiotepa/carmustine or TBC) for older PCNSL patients aged ≥65y, the BCNU/Thio group was associated with a lower risk for NRM alongside improved PFS and OS [105]. According to another retrospective, multicenter study, HD-AraC-containing treatment regimens and consolidation with HD-BCU-TT/ASCT or WBRT were associated with superior survival in PCNSL patients treated with HD-MTX-based induction therapies [106]. However, in a randomized phase 2 clinical trial to compare myeloablative consolidation (thiotepa + carmustine) supported by ASCT with nonmyeloablative consolidation (etoposide + cytarabine) after induction therapy for PCNSL, the results revealed that the estimated 2-year PFS rate in those who completed consolidation therapy was not significantly different (86% vs. 71%; *p* > 0.05) [107].

A maintenance therapy for PCNSL has yet to be established. Some studies suggested that low-dose lenalidomide (LEN, 5–10 mg/dose on a 21-day cycle) as a maintenance therapy after induction chemotherapy in older PCNSL was well tolerated with an excellent PFS and OS (with an overall median follow-up of 31.6 months; median mPFS and mOS had not been reached) [108]. However, a recent retrospective study showed that the first-line LEN maintenance treatment did not improve PFS and OS, although the safety was satisfactory in patients with PCNSL [109]. From a Mayo Clinic experience, HD-MTX maintenance therapy followed by HD-MTX-based induction therapy might be a reasonable treatment strategy for PCNSL. At a median follow-up time of 4.5 years, the PFS rate in the HD-MTX maintenance cohort was 72.6% with an OS rate of 82.4%, which was not significantly different compared to the ASCT cohort (74.6% and 76.0%, respectively) [110]. In some studies, the OS of HD-MTX maintenance was better than WBRT after the induction of HD-MTX-based chemotherapy [111]. A phase 2 prospective study indicated that ibrutinib maintenance (560 mg/d) should be a well-tolerated and effective treatment in elderly PCNSL. The results showed that the 2-year PFS rate is 72.6%, and the 2-year OS rate is 89% [112]. In a 10-year follow-up of the Nordic phase II study, 10-year OS rate with the first-line maintenance TMZ for PCNSL was 29.8% [113]. However, another study failed to demonstrate the benefit of TMZ maintenance [114].

### 5.3. Treatment of Recurrent/Refractory (R/R) PCNSL

About 10–25% of patients have no response to chemotherapy, and 25–50% of them relapse after an initial response, with a higher rate in older patients [17,61]. Refractory PCNSL in patients is defined as a response rate less than PR or by the persistence of abnormal CSF after induction treatment. There is no consensus on the treatment for R/R PCNSL. Further treatment should be weighed against age, clinical conditions, prior treatments, and overall response. For R/R PCNSL, potential options include immunotherapy, new drugs, or participation in clinical trials, such as anti-CD20 monoclonal antibody (i.e., rituximab), Bruton’s tyrosine kinase (BTK) inhibitor, immunomodulatory drugs (IMIDs), etc. [115]. If relapse occurs after >1 year, another course of HD-MTX can be used. ASCT or WBRT can also be considered if the patient did not receive the therapy in earlier treatment [62].

#### 5.3.1. Immunotherapy and Novel Targeted Treatments

There is currently no standard treatment for R/R PCNSL. Anti-CD20 monoclonal antibody, BTK inhibitors, and IMIDs have been reported for second-line treatments in R/R PCNSL.

a. - Novel monoclonal antibodies and bispecific antibodies

Rituximab, the anti-CD20 monoclonal antibody (mAb), is widely used in DLBCL as a first-line standard therapy. Considering that most PCNSL are subtypes of DLBCL, rituximab has been integrated into the treatment of PCNSL patients (i.e., MATRix). Novel monoclonal antibodies (i.e., anti-CD79b monoclonal antibody, Loncastuximab [116]) and bispecific antibodies (BsAb, i.e., anti-CD20xCD3, anti-CD19xCD3) showed various response rates in R/R LBCL and CNSL clinical trials [117,118,119]. These results suggest that novel mAb/BsAb may be an effective therapy against PCNSL, but data are insufficient.

b. - BTK inhibitors

Using WES, the majority of PCNSL samples have been classified as the MCD type [2], relying on sustained activation of the BCR signaling pathway to benefit from the treatment of BTK inhibitors, which could readily cross the BBB [12,120]. In a phase 1b trial of ibrutinib/HD-MTX/rituximab combination therapy in R/R PCNSL, clinical responses occurred in 80% of patients [121]. In a prospective, multicenter, phase II study, the mPFS of ibrutinib in R/R PCNSL or primary vitreoretinal lymphoma (PVRL) was 4.8 months, while the mOS was 19.2 months [122]. Tirabrutinib, a second-generation BTK inhibitor, showed favorable efficacy in R/R PCNSL with an ORR of 64% and mPFS of 2.9 months [123]. According to previous and ongoing clinical trials, first- and second-generation BTK inhibitors (including zanubrutinib and orelabrutinib) have been shown to be effective treatment options for R/R PCNSL, even for newly diagnosed PCNSL patients [124].

c. - IMIDs

In a phase II study, the ORR of LEN combined with rituximab was 35.6% in R/R PCNSL, with an mPFS of 7.8 months and an mOS of 17.7 months [125]. Pomalidomide, a third-generation IMID, combined with dexamethasone, showed significant therapeutic activity in R/R PCNSL and PVRL, with an ORR of 50%, in a phase 1 study at the Mayo Clinic [126]. The efficacy of the combination of pomalidomide/orelabrutinib/rituximab (POR regimen) in a phase II, single-arm trial for a newly diagnosed PCNSL (NCT05390749) and the combination of pomalidomide + thiotepa for R/R PCNSL (NCT05931328) are being evaluated in ongoing studies. The results showed that the ORR of POR was 90.1%, which was reported during the 2023 EHA.

d. - Chimeric antigen receptor T-cell (CAR-T) therapy

CD19-targeted CAR-T therapy is considered a revolutionary treatment for large B-cell lymphoma (LBCL). Recently, CAR-T-cell therapy has shown promising efficacy in PCNSL [127]. Based on a phase 1/2 clinical trial of R/R PCNSL, 58.3% of patients achieved a response when treated with tisagenlecleucel CAR-T-cells, with a CRR of 50% [128]. In a meta-analysis of 128 patients, 56% of PCNSL patients achieved CR, with 37% remaining in remission after 6 months [129]. In a retrospective study of relapsed PCNSL, the results showed that CAR-T-cell therapy (tisa-cel and axi-cel) achieved a CRR of 64% with a 43% 1-year PFS rate and a 79% 1-year relapse-free survival (RFS) rate [130]. In another retrospective study of CD19/CD22 CAR-T-cell therapy following ASCT in R/R PCNSL, the ASCT + CAR-T group had a higher ORR and 2-year PFS rate compared with the chemoimmunotherapy group (82.75% vs. 58.83%, 65.52% vs. 30.00%, respectively) [131]. Based on these outcomes, CAR-T therapy might become a preferred treatment for R/R PCNSL. In a multicenter retrospective analysis of CAR-T (commercial relma-cel) used in R/R CNSL patients (PCNSL=12, SCNSL=10), the best ORR was 90.9%, with an estimated 1-year PFS rate, DOR, and OS rate of 64.4%, 71.5%, and 79.2%, respectively. Furthermore, the results also proved the benefit of a BTK or PD-1 inhibitor on CAR-T-cell re-expansion, which might suggest novel dual-agent CAR-T-related combinatorial therapies [132]. Moreover, recent studies demonstrate that the combination of CAR-T and WBRT could be a promising therapy for R/R CNS-B-cell lymphoma patients, associated with improving remission rates and safety [133,134].

#### 5.3.2. Other Novel Targeted Treatments 

Nayak et al. reported that PD-1 inhibitor (Nivolumab) had clinical responses in five patients with PCNSL or SCNSL, and three patients remained progression-free at 13+ to 17+ months [135]. According to a meta-analysis, the pooled ORR of patients treated with PD-1 inhibitor (nivolumab or pembrolizumab) was 67.1%, and CRR was 42.8%, but with high heterogeneity. The 6-month PFS rate was 34.8% with low heterogeneity [136]. Some studies showed that PI3K/AKT/mTOR signaling pathways were activated in methotrexate-resistant PCNSL-derived cells [137]. Temsirolimus, an mTOR inhibitor, was found to be effective in R/R PCNSL with an ORR of 54%, an mPFS of 2.1 months, and an mOS of 3.7 months. However, the responses were short-lived [138]. However, PI3K inhibitor Buparlisib monotherapy showed no meaningful responses in PCNSL based on a clinical trial [139]. The previous studies proved that human PCNSL had high expression of interleukin-1 receptor-associated kinase-4 (IRAK-4), and IRAK-4 might be a novel target of PCNSL [140]. A phase 1/2 clinical trial evaluating the IRAK-4 inhibitor emavusertib (CA-4948) combined with ibrutinib in treating R/R hematologic malignancies including PCNSL (NCT03328078) is ongoing. In a case report, Zou R et al. reported a patient with multiline-resistant refractory PCNSL, who received a CD19/CD22 dual-targeted CAR-T, PD-1, and BTK inhibitor combination and maintained a CR for 35 months [141]. In some experimental studies, other small-molecule drugs penetrating BBB (like Selinexor [142,143] and venetoclax [144]) might also show efficacy in PCNSL, but clinical data are lacking. A dual PI3K/HDAC inhibitor, BEBT-908, showed anti-tumor activity in brain orthotopic lymphoma mouse models [145]. These studies show novel potential in treatments of PCNSL.

**Figure 3:**
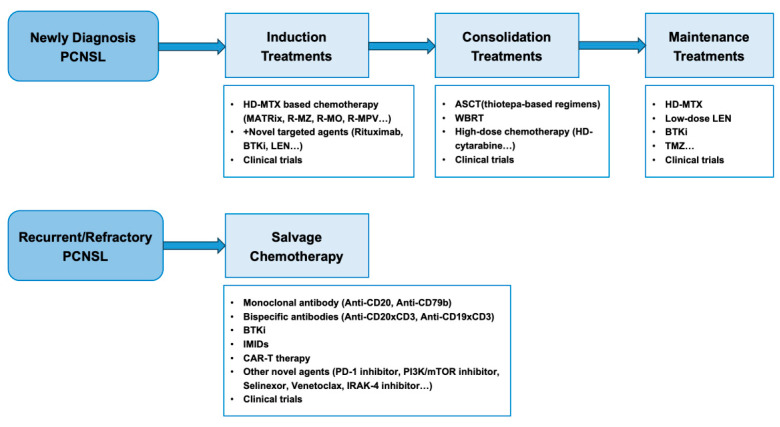
The treatment strategies for PCNSL.

**Figure 4:**
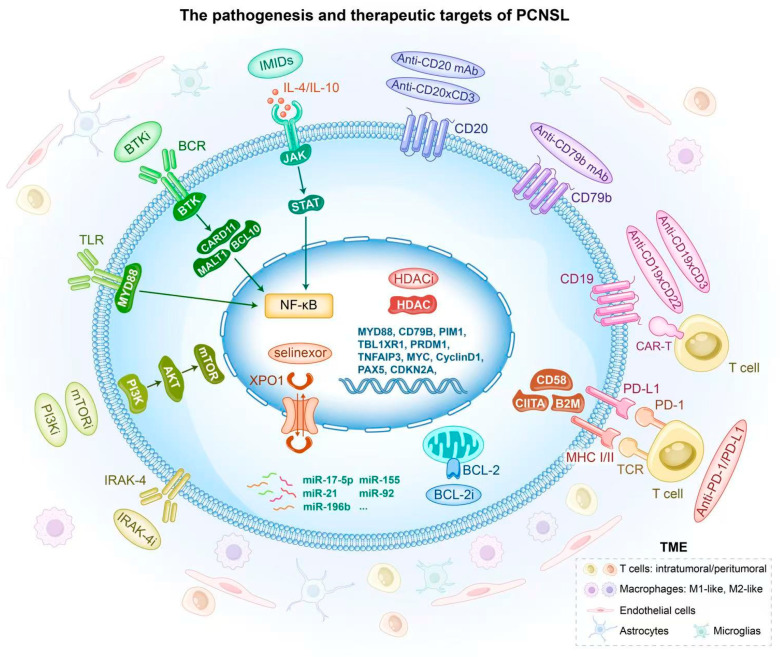
The pathogenesis and therapeutic targets of PCNSL. Molecular pathways and genomic aberrations in the pathogenesis of PCNSL including NF-κB, TLR, BCR, JAK-STAT, and PI3K-mTOR signaling. The heterogeneous TME and its related genes also play an important role in the pathogenesis of PCNSL. Recent novel therapies, including novel monoclonal antibodies, bispecific antibodies, BTK inhibitors, immunomodulatory drugs, immune checkpoint inhibitors, PI3K/mTOR inhibitors, and chimeric antigen receptor (CAR) T-cell therapy, have demonstrated great potential in the treatment of PCNSL.

The treatment strategies for PCNSL are illustrated in Figure 3. The molecular pathways and genomic aberrations in the pathogenesis of PCNSL and corresponding therapies are illustrated in Figure 4.

## 6. Conclusion and Future Directions

PCNSL is a relatively rare, aggressive extra-nodal non-Hodgkin lymphoma with distinct clinicopathological and genetic characteristics. Progress in neuroimaging and radiomics will improve the accuracy of early diagnosis. Brain biopsy by stereotactic biopsy remains the gold standard for diagnosis of PCNSL. In patients unsuitable for biopsy, CSF detections can be used to facilitate the diagnosis of PCNSL, including cytology, FCM, ctDNA, and cytokine concentration tests. The widespread use of WGS integrated with transcriptome sequencing helps us to illuminate the role and mechanisms of coding and non-coding mutations, while the development of spatial single-cell transcriptome analyses has led to a better understanding of the TME in PCNSL. The use of novel agents, including BTK inhibitors, IMIDs, immune checkpoint inhibitors, other signaling pathway inhibitors, and CAR-T therapy, has shown great potential in improving the poor outcomes in relapsed/refractory disease. The further identification of molecular events involved in the pathophysiology of PCNSL will promote the development of novel treatments with greater effectiveness and lower toxicity. Patients will also benefit more from emerging agents that are tailored for individual TME states and agents that enhance BBB permeability.

## Abbreviations

The following abbreviations are used in this manuscript:

PCNSL	Primary Central Nervous System Lymphoma
CNS	Central Nervous System
GCB	Germinal Center B-cell-like
WHO	World Health Organization
EBV	Epstein–Barr Virus
BBB	Blood–brain Barrier
ABC	Activated B-Cell-like
CNVs	Copy Number Alterations
TME	Tumor Microenvironment
CS	Significant Cluster
IME	Invasive Margin Excluded
IMS	Invasive Margin immunosuppressed
CT	Computed Tomography
MRI	Magnetic Resonance Imaging
FDG	Fluorodeoxyglucose
PET	Positron Emission Tomography
IPCG	International Primary CNS Lymphoma Collaborative Group
SCNSL	Secondary Central Nervous System Lymphoma
CSF	Cerebrospinal Fluid
PD-L1	Programmed Death Ligand 1
FCM	Flow Cytometry
ctDNA	circulating tumor DNA
miRNA	microRNAs
IL-10	Interleukin-10
OS	Overall Survival
HD-MTX	High-dose Methotrexate
ASCT	Autologous Stem Cell Transplant
WBRT	Whole Brain Radiotherapy
EANO	European Association of Neuro-Oncology
TMZ	Temozolomide
ORR	Overall Response Rate
CR	Complete Remission
RCT	Randomized Controlled Trials
PR	Partial Response
NRM	Non-Relapse Mortality
LEN	low-dose lenalidomide
BTK	Bruton’s Tyrosine Kinase
IMIDs	Immunomodulatory Drugs
BsAb	Bispecific Antibodies
PVRL	Primary Vitreoretinal Lymphoma
POR	Pomalidomide/Orelabrutinib/Rituximab
CAR-T	Chimeric Antigen Receptor T cell
LBCL	Large B-Cell Lymphoma
RFS	Relapse-free Survival
IRAK-4	Interleukin-1 Receptor-associated Kinase-4
WGS	Whole-genome Sequencing
